# Tristetraprolin promotes survival of mammary progenitor cells by restraining TNFα levels

**DOI:** 10.3389/fcell.2023.1265475

**Published:** 2024-01-11

**Authors:** Micaela Stedile, Angela Lara Montero, Martín Emilio García Solá, María Victoria Goddio, Inés Beckerman, Emilia Bogni, Marina Ayre, Zaira Naguila, Omar A. Coso, Edith C. Kordon

**Affiliations:** ^1^ Instituto de Fisiología, Biología Molecular y Neurociencias, Universidad de Buenos Aires, Consejo Nacional de Investigaciones Científicas y Tecnológicas (IFIBYNE-UBA-CONICET), Ciudad de Buenos Aires, Argentina; ^2^ Facultad de Ciencias Exactas y Naturales (FCEN), Universidad de Buenos Aires (UBA), Ciudad de Buenos Aires, Argentina; ^3^ Departamento de Fisiología, Facultad de Ciencias Exactas y Naturales (FCEN), Universidad de Buenos Aires (UBA), Ciudad de Buenos Aires, Argentina; ^4^ Departamento de Química Biológica (DQB), Facultad de Ciencias Exactas y Naturales (FCEN), Universidad de Buenos Aires (UBA), Ciudad de Buenos Aires, Argentina

**Keywords:** mammary gland, TTP, mammary stem/progenitor cells, MAPK-p38, TNFα

## Abstract

Tristetraprolin (TTP) is an RNA binding protein that destabilizes mRNAs of factors involved in proliferation, invasiveness, and inflammation. Disruption of the gene that codes for TTP (*Zfp36*) led to severe arthritis, autoimmunity, cachexia and dermatitis in mice. It has been shown that these phenotypes were mostly due to excessive TNFα levels in the affected tissues. We have previously reported that TTP expression is required for lactation maintenance. Our results indicated that conditional MG TTP-KO female mice displayed early involution due to the untimely induction of pro-inflammatory pathways led mostly by TNFα overexpression. Here we show that reducing TTP levels not only affects the fully differentiated mammary gland, but also harms morphogenesis of this tissue by impairing the progenitor cell population. We found that *Zfp36* expression is linked to mammary stemness in human and mice. In addition, diminishing TTP expression and activity induced apoptosis of stem-like mouse mammary cells, reduced its ability to form mammospheres in culture and to develop into complete glands when implanted into cleared mammary fat pads *in vivo*. Our results show that survival of the stem-like cells is compromised by increased levels of inflammatory cytokines and stimulation of signaling cascades involving NFκB, STAT3 and MAPK-p38 activation. Moreover, TNFα overexpression and the consequent p38 phosphorylation would be the leading cause of progenitor cell death upon TTP expression restriction. Taken together, our results reveal the relevance of TTP for the maintenance of the mammary progenitor cell compartment by maintaining local TNFα levels at bay.

## 1 Introduction

Tristetraprolin (TTP) is a protein, encoded by the *Zfp36* gene, that negatively regulates expression of multiple targets involved in inflammation, cell proliferation, angiogenesis, epithelial to mesenchymal transition and invasiveness. Typically, TTP binding to the 3′untranslated regions (3′UTR) of specific mRNAs leads to their destabilization, although this protein can also exerts transcriptional modulation of various genes (Guo et al., 2017; Kovarik et al., 2021). Importantly, it has been reported that TTP-KO mice showed a dramatic systemic inflammatory syndrome due, at least partly, to the overexpression of TNFα. On the other hand, TTP overexpression protected against the development of inflammatory diseases in various experimental models (Patial and Blackshear, 2016).

Mastitis, an inflammation of the mammary gland caused by infection or autoimmunity, has a relevant impact in women of fertile age (Goulabchand et al., 2020). It has been shown that TNF-α inhibitors may be a potential treatment for at least some types of this disease (Chiu et al., 2022). We have previously reported that TTP is expressed in the mammary differentiated epithelium (Goddio et al., 2012) and that its presence is required for lactation maintenance in the female mice. We found that lactating TTP^fl/fl^ x Wap-Cre bitransgenic females (MG TTP-KO) showed premature involution due, at least partly, to excessive levels of TNFα in the mammary tissue (Goddio et al., 2018).

The mammary epithelium entails two main cellular lineages: luminal cells that border the ductal and alveolar lumen, and myoepithelial or basal cells, which are located next to the basement membrane (Watson and Khaled, 2008). The proliferative changes within this epithelium are fueled by embryonic pluripotent mammary stem cells (MaSCs), capable of originating both basal and luminal lineages, and by unipotent progenitor cells (either luminal or basal) that amplify and maintain each of these compartments in the post-natal gland throughout the successive reproductive cycles of the adult female mouse (Visvader and Stingl, 2014). Starting at puberty, a tree of branching ducts driven by the terminal end buds (TEBs) located at their ends, advance until they reach the limits of the mammary fat pad, when these structures disappear (Williams and Daniel, 1983). In each pregnancy, progenitor cells promote the development of the secretory lobuloalveolar compartment, which is the main source of milk production during lactation. After weaning, these differentiated structures regress due to massive cell death and tissue remodeling. This involution process is associated with inflammatory pathways that involve activation of transcription factors, such as STAT3 and NFκB (Watson and Kreuzaler, 2011; Inman et al., 2015). However, a pregnancy-induced mammary epithelial cell (PI-MEC) population that originates in the first pregnancy and lactation cycle do not die-out during involution and persists throughout the rest of the female mouse life. These cells possess stem cell-like features, are at least partially responsible for subsequent lobular development and may behave as cancer stem cells (Wagner and Smith, 2005).

Here we show that TTP downregulation affects not only the fully differentiated mouse mammary epithelium, but also the progenitor cell population. We found that this deleterious effect was mostly due to the dramatic overexpression of TNFα that led to p38-dependent cell death. Therefore, these results suggest that inflammatory diseases of the mammary gland may have an impact on lactation success in following reproductive cycles.

## 2 Materials and methods

### 2.1 Mouse models

Mice were maintained in a pathogen-free facility at constant temperature of 22°C ± 2°C and 40%–70% humidity in a 12-h light cycle with food and water *ad libitum*. Wap-Cre/TTP^fl/fl^ (MG TTP-KO) strain was obtained by crossing TTP^fl/fl^ with Wap-Cre mice, both in C57BL/6 genetic background (Goddio et al., 2018). TTP^fl/fl^ mice, generated and provided by Dr. Perry Blackshear´s lab (NIEHS, NIH, USA), have exon 2 of *Zfp36* surrounded by LoxP sequences (Qiu et al., 2012). The Wap-Cre mice were obtained from the NCI-NIH Mouse Repository, United States. Genomic DNA was obtained from mouse tails by “HotSHOT,” as previously described (Truett et al., 2000). Wap-Cre/TTP^fl/fl^ mice were identified by PCR. *Zfp36* floxed and *Zfp36* WT alleles were amplified with P1_Fw and P2_Rv primers. Wap-Cre allele was amplified with W003_Fw and C031_Rv primers (see Supplementary Table S1). Removal of *Zfp36* exon 2 in MG-TTP KO mammary glands was tested by multiplex PCR with two forward primers (P1_Fw, P3_Fw) and reverse primer P4_Rv. Product size of each PCR is shown in Supplementary Table S2 and corresponding agarose gel images are shown in Supplementary Figure S5.

MMTV-Cre mice (from the NCI-NIH mouse repository) were crossed with TTP^fl/fl^ mice. Bitransgenic female mice were identified by PCR as described above. MMTV-Cre allele was amplified using MMTV-Cre_Fw and CRE_C031_RV primers (Supplementary Table S1). Product size is shown in Supplementary Table S2. KO of TTP was checked by multiplex PCR as described above (Supplementary Figure S5). Eight-week-old females were euthanized and whole mount analysis of their mammary glands were performed.

Inguinal mammary fat pads of 21-day-old C57BL/6 (for TTP-KO tissue implants) or BALB/c (for HC11 cell injection) female mice were cleared of endogenous epithelium to perform implantation studies as described by Blair and DeOme, 1961. For TTP-KD or Sh-Ctrl HC11 cells, 1.0 × 10^5^ and 2.0 × 10^5^ cells were implanted in 100 µl. After 10 weeks, recipient mice were euthanized and mammary whole mounts were performed. Tertiary ducts, which originate at second branching events (Macias and Hinck, 2012; Huebner and Ewald, 2014), were analyzed in quantity and length.

### 2.2 Ethics statement

Mouse experiments were approved by local IACUC authorities (Comisión Institucional para el Cuidado y Uso de Animales de Laboratorio–CICUAL FCEN-UBA) and complied with regulatory standards of animal ethics (ARRIVE guidelines, Rs principles and AVMA guidelines).

### 2.3 Monolayer cell culture, transfections and treatments

HC11 mouse mammary cell line and derived sublines were cultured at 37°C and 5% CO_2_ in RPMI with HEPES (Gibco, #23400021) supplemented with 5 μg/mL insulin, 1% antibiotic antimycotic (Gibco, #15240062) and 10% Fetal Bovine Serum (Internegocios, #FBI). The TTP-KD HC11 subline was initiated by transfecting *Zfp36* sh-RNAs (Mission-Merk TRCN0000238325, TRCN0000238326, TRCN0000238328) cloned into pKLO.1-Puro vectors. These vectors were transfected with PEI (Polysciences), using 5 μg of total DNA in 6-well plates, (PEI: DNA 3:1). Clones were selected with 3 μg/mL (and maintained with 1 μg/mL) of Puromycin (Invivogen, #ant-pr-5b).

Plasmids pTRE2hygLUC-3′UTR-*Tnfα* and pCMVLacZ were transfected with Lipofectamine 3000 (Thermo, #L3000001) using 1 μL/μg DNA, P3000 Reagent at 2 μL/μg DNA, and opti-MEM (Gibco, #A4124801). To harvest cells and perform luciferase readings, Reporter Lysis Buffer (Promega, #E1531) was employed.

For inhibiting MAPKs p38, ERK1/2 and JNK1/2, 10 μM SB203580, 20 µM PD98059 and 10 µM SP600125 (Calbiochem #559389, #513000 and #420119) or vehicle (DMSO) were used in medium with no FBS for 2 h.

### 2.4 RT-qPCR assays

Monolayer cultures were harvested with RNA-PrepZOL (Inbiohighway, #R0010) according to manufacturer instructions. RNA was reverse transcribed (RT) using MMLV InbioHighway (#E1601) reagent. All qPCRs were performed using SYBER Green (Roche) in the StepOnePlus equipment (Thermo). Gene expression levels were normalized to *RNA 18s* using standard curves (sequences in Supplementary Table S3).

### 2.5 Cell viability and apoptosis assays

We used the CellTiter 96^®^ AQueous One Solution Cell Proliferation Assay (Promega, #G3582) for determining number of viable cells. Calibration curves were made by adding MTS 24 h post-plating and measuring the absorbance at 490 nm between 2.5 and 3.5 h later.

Apoptotic cells were detected using the DeadEnd™ Colorimetric TUNEL System (Promega, #G7360). Diaminobenzidine (DAB) was used as chromogen and hematoxylin as counterstaining. At least four fields per condition were photographed and quantified. For positive control, cells were treated with DNase I (4u/mL) for 10 min.

### 2.6 Primary cultures

Thoracic, abdominal and inguinal mammary glands were obtained with sterilized surgery tools and mechanically dissected in RPMI supplemented with HEPES, penicillin/streptomycin 1% v/v, 0.15% collagenase type 4, 0.2% trypsin, DNase 10 μg/mL and 2% FBS (Fetal Bovine Serum). Then, they were incubated in agitation for 1 h at 37°C and centrifuged several times to remove adipocytes from mammary epithelial cell pellets, which were resuspended in RPMI supplemented with HEPES, penicillin/streptomycin 1% v/v and 10% FBS. Cells were plated and monitored for up to 4 days, when they were trypsinized and used for mammosphere assays.

### 2.7 Mammosphere assays

Ultra-Low Attachment 6-well plates (Corning, # CLS3471-24EA) were used. Cells from primary cultures were plated at low density (10,000 cells/mL) in RPMI plus HEPES media supplemented with 1% antibiotic/antimycotic (Gibco #15240062), 5 μg/mL insulin, 20 ng/mL human recombinant EGF (Thermo #PHG0313) and 1X Gem21 NeuroPlex™ Serum-Free (B27) (GeminiBio, #400-160). No serum was added in these assays. In those involving TTP-KD or Sh-Ctrl cells, 1 μg/mL Puromycin was added. Mammosphere formation was assessed 10 days post-plating by taking photos of 5–10 fields per condition and analyzed them by ImageJ. MAPK p38 was inhibited and TNFα was blocked by adding either 10 µM SB203580 or 0.03 μg/μL Etanercept for 8–10 days.

### 2.8 Western blot (WB) analysis

Cells were harvested in RIPA buffer with 1:100 protease inhibitor cocktail set I (Calbiochem, #539131) and phosphatase inhibitors (1:100 Na_2_Ov_4_; 1:20 NaF). Most membranes were blocked for 1 h in 5% milk, but those for detection of phosphorylated proteins were blocked overnight in 5% gel fish (Sigma, #G7765). Blots were tested with antibodies listed in Supplementary Table S4. For MAPK analysis, fluorescent secondary antibodies were used and detected with the Odyssey imaging system. For studying other proteins, horseradish peroxidase (HRP) conjugated secondary antibodies were utilized and detection was performed with Amersham ImageQuant 800 equipment.

### 2.9 Immunofluorescence (IF)

For mammary gland and cell culture immunofluorescence, fixation was carried out in 4% paraformaldehyde (PFA), tissue was then included in *OCT* compound, frozen, and sectioned in 20 µm slides by cryostat. For DNA staining, slides were incubated with RNase A (10 μg/mL) for 3 h at 37°C and 1 μg/mL propidium iodide (PI) was added for 5 min. Primary and secondary antibodies are listed in Supplementary Table S5.

### 2.10 Immunohistochemistry (IHC)

Mouse tissues were formalin-fixed, paraffin-embedded, and sectioned. After sample dewaxing and rehydration, antigen retrieval was performed with 10 mM Citrate buffer pH 6.0 for 10 min at sub-boiling tempeture. Sections were exposed for 2 min to 2% H2O2 and, after washing, blocked for 1 h in 2.5% bovine serum albumin (BSA). The samples were incubated overnight at 4°C with the following antibodies diluted in 2.5% BSA: anti-CC3 (#9661, Cell Signaling Technology, 1:200), and anti-ZFP36/Tristetraprolin (#C431045, LSBio, 1:200). The next day, after washing, the samples were incubated for 1 h with an anti-rabbit biotinylated antibody, diluted 1:400 in 2.5% BSA. Next, samples were washed and incubated with the ABC reagent (PK-6101, Vector Laboratories) for 30 min. Finally, the sections were washed and stained with the DakoCytomation LSAB + System-HRP (Dako). Sections were counter-stained with hematoxylin. For CC3 and ZFP36/Tristetraprolin-staining, pictures (200X and 400X, respectively) covering the whole area of the gland were taken. For CC3 quantification was done using ImageJ software and expressed as the number of positive CC3 cells/1,000 cells ±S.D. In the figure legends, the magnification of the pictures is indicated by scale bars.

### 2.11 Mammary whole mounts (WM)

Whole mammary glands were extended on slides and fixed for 2 h in Carnoy’s solution (6:3:1 Ethanol: Chlorophorm: Glacial acetic acid). Tissue was hydrated to be stained O.N. with carmine-alum, dehydrated and mounted.

### 2.12 In silico analysis

Analysis of gene expression throughout different molecular subtypes of breast cancer were performed using The Cancer Genome Atlas (TCGA) -Pan-Cancer database and the METABRIC dataset. To evaluate expression levels differences, Kruskal Wallis and Dunn tests were carried out. To determine correlations, Spearman’s ranked correlation test was used. For scRNA-seq analysis, matrices from Bach et al., 2017, Giraddi et al., 2018; Pal et al., 2017 were downloaded and loaded into R, version 3.6.3. The data corresponding to adult mice mammary samples were integrated into a single matrix and analyzed using the Seurat R package, version 3.4.4. A UMAP was generated using the top 3,000 variable genes selected via the default Variance Stabilizing Transformation (VST) method. Cluster/cell type labels were preserved from the original manuscripts for visualization and downstream analysis. Specific details in García Solá et al., 2021 and https://github.com/martings/JOMG-Garcia_Sola_et_al.

### 2.13 Statistical tests

Statistical significance of differences was evaluated using GraphPad Prism8. Data was presented as mean ± SEM unless otherwise noted. In every case, at least three independent experiments were evaluated.

### 2.14 Flow cytiometry

Lymph node–free mammary glands of 4 TTPfl/fl and 4 Wap-CRE x TTPfl/fl 10 females were taken 10 days after natural weaning (20 days after delivery). Tissue was minced and digested to obtain single-cell suspensions. For FACS, 5 × 10^5^ cells were stained with fluorophore-conjugated antibodies (BioLegend). Specifically, cells were stained with anti-mouse CD31/CD140a/TER119/CD45-PE (Lineage cocktail; clones 145-2C11, RB6-8C5, M1/70, RA3-6B2, and Ter-119), anti-hamster IgG/anti-rat IgG2b/anti-rat IgG2a as lineage cocktail isotype controls, anti-mouse CD24-APC (clone M1/69), or anti-mouse CD29-FITC, for 30 min on ice in 10% (v/v) FBS/PBS. Stained populations were analyzed and sorted on a BD FACS Aria (II) Cell Sorting System (BD). Data acquisition and analysis were performed using Flow Jo V10 software.

## 3 Results

### 3.1 TTP/*Zfp36* downregulation induces mammary progenitor cell impairment *in vivo*

**FIGURE 1:**
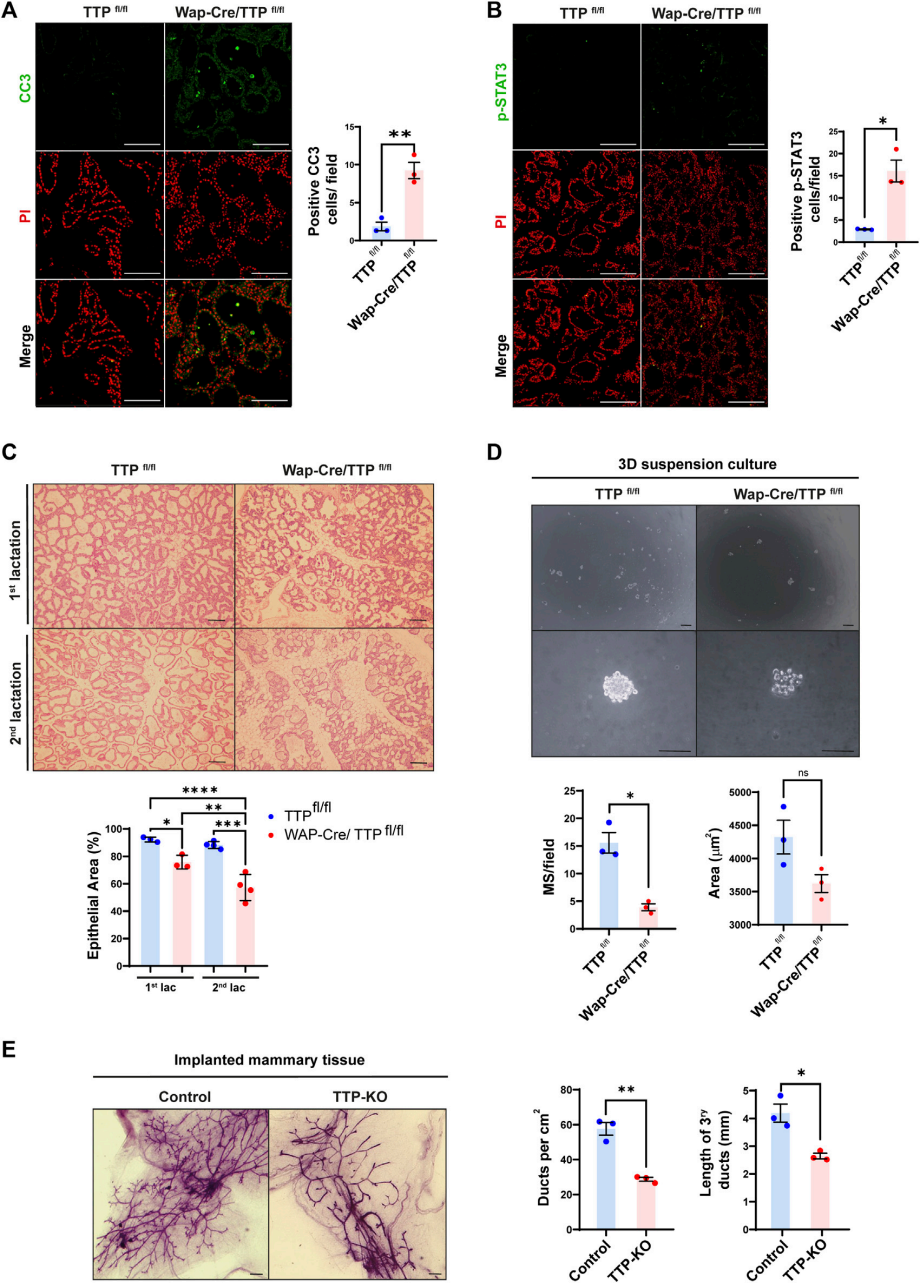
Phenotype of mammary glands of MG TTP-KO and total TTP-KO mice. **(A, B)** Representative images of immunofluorescence (IF) assays for CC3 **(A)** and for p-Stat3 **(B)** of Wap-Cre/TTP^fl/fl^ (MG TTP-KO) females and TTP^fl/fl^ (control) mice at day 15 of their second lactation. Nuclei were stained with propidium iodide (PI). Quantification plots of positive nuclei per field ±SEM are shown on the right (T-Student test, *n* = 3, *p < 0.05, **p < 0.01). Scale bar = 100µm; OM = 200X. **(C)** Representative images of H&E-stained mammary glands from TTP^fl/fl^ and Wap-Cre/TTP^fl/fl^ (MG TTP-KO) during first and second lactation. OM = 100X. Scale bars = 100µm. Below, quantification plots show % of area occupied by epithelium ±SEM (One-way ANOVA and Tukey test, *n* = 3–4, significant differences are represented by asterisks (*p ≤ 0.05, **p ≤ 0.01, ***p ≤ 0.001, ****p ≤ 0.0001). **(D)** Representative light field images of mammospheres (MS) at 10 days post-seeding derived from MECs of post-involuting TTP^fl/fl^ and Wap-Cre/TTP^fl/fl^ mammary glands (upper panels: OM = 40X; lower panels: OM = 400X; scale bar = 100 µm). Below, quantification plots of average number of MS per field ±SEM and average area occupied by each MS ± SEM (T-Student test, *p < 0.05, ns = non-significant). Each dot corresponds to one mouse from which mammary cells were transferred to the plates. **(E)** Representative images of whole mounted mammary glands from TTP-KO or control (C57BL/6 WT mice) implants into cleared fat pads of syngeneic female mice. Scale bars = 1 mm. On the right, quantification plots of average ducts per cm^2^ ± SEM and tertiary duct length ±SEM (T-Student test, *n* = 3, *p < 0.05, **p < 0.01). OM: Original Magnification.

WAP gene promoter allows the expression of a transgene specifically in the mammary gland during lactation (Andres et al., 1987). Interestingly, throughout that period, in addition to the secretory cells, the WAP promoter is active in a progenitor compartment, the parity-induced mammary epithelial cells (PI-MEC), which are able to proliferate and produce new secretory acini in successive pregnancies (Boulanger et al., 2005). Therefore, to determine the impact of TTP expression impairment in progenitor cells using TTP^fl/fl^ x Wap-Cre bitransgenic females (MG TTP-KO), we analyzed the mammary tissue at the second lactation period. As previously observed during first lactation (Goddio et al., 2018), MG TTP-KO mammary glands displayed signs of premature involution, like significant increase of cleaved caspase 3 (CC3), STAT-3 phosphorylation (p-STAT3, Y705) (Figures 1A, B) and lighter litters than those from control mice (Supplementary Figure S1A) at day 15 post-delivery. In addition, we observed that developmental deficiency of MG TTP-KO mammary glands was greater in the second reproductive cycle compared with the first one (Figure 1C). Apoptosis increase in mammary glands of MG TTP-KO was also detected during the second pregnancy of these mice (Supplementary Figure S1B).

To confirm the impairment of the progenitor mammary cell compartment, primary cultures of post-involuting glands were carried out. We found that MG TTP-KO cells took longer to attach and grow than cells from control mice (Supplementary Figure S1C), and that when plated on ultra-low attachment plates, they formed less mammospheres than their control counterparts (Figure 1D). These data suggested that survival of WAP-expresser PI-MECs has been negatively affected by TTP loss. In addition, flow cytometry assay of post involuting MG-TTP KO and control mammary glands revealed that the former displayed an important deficit of mammary epithelial cells, including in the basal/progenitor compartment (Supplementary Figure S1D).

To verify whether mammary progenitor compartments may be altered in other mouse models with TTP deficiency, we implanted mammary tissue fragments from total TTP-KO or C57BL/6 wild type (control) mice into inguinal cleared fat pads of 3-week-old syngeneic females. After 10–12 weeks, whole mount analysis revealed that mammary glands from TTP-KO implants developed significantly lower number and length of tertiary ducts (Figure 1E). Similarly, mammary glands of 8–10 weeks-old MMTV-Cre x TTP^fl/fl^ females displayed fewer major and branching ducts as well as a higher number of remaining TEBs, associated to incomplete invasion of cleared fat pads, than TTP^fl/fl^ control mice (Supplementary Figure S1E).

### 3.2 Bio-informatic analysis of TTP/*Zfp36* expression profile in mouse mammary gland and breast cancer data sets

**FIGURE 2:**
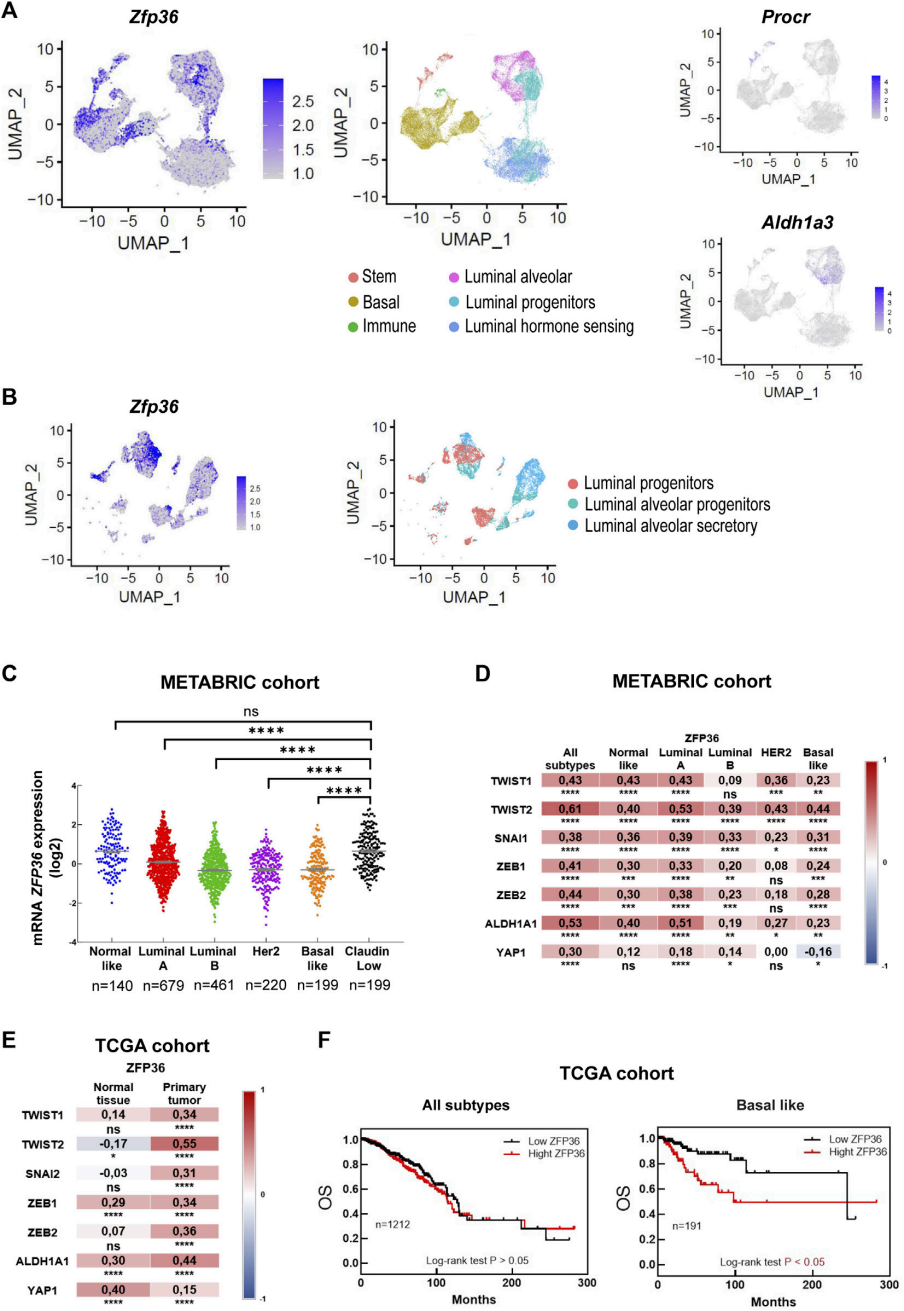
Bioinformatic TTP/*Zfp36* expression analysis in mouse mammary epithelial cells and human breast tissue. **(A)** Uniform Manifold Approximation and Projection (UMAP) plot of postnatal MEC populations displaying scRNAseq relative expression levels of *Zfp36, Procr and Aldh1a3* in color intensity gradient. **(B)** UMAP plot of mammary luminal cell populations displaying relative expression levels of *Zfp36,* in color intensity gradient. Cell populations, as previously identified by García Solá et al., 2021, are indicated by color patterns. **(C)** Expression of *Z* *FP36* mRNA (log2 values) in breast cancer molecular subtypes according to METABRIC database. Kruskal Wallis and Dunn’s tests were performed. Significant differences are represented by asterisks (*p ≤ 0.05, **p ≤ 0.01, ***p ≤ 0.001, ****p ≤ 0.0001). Number of patients (n) is indicated below each plot. **(D, E)** Expression correlation between *Z* *FP36* and genes related to stem/progenitor behavior comparing different breast cancer subtypes according to METABRIC database **(D)**, and between normal tissue and primary tumors according to TCGA data **(E)**. On the right of each graph, color scale (red: positive, blue: negative) indicates correlation level. Spearman’s ranked correlation test was applied (*p < 0.05, **p < 0.01, ***p < 0.001, ****p < 0.0001). **(F)** Overall survival (OS) of breast cancer patients (all subtypes, on the left; basal-like subtype, on the right) from TCGA database categorized according to *ZFP36* expression levels. Number of patients (n) is indicated in each graph. To test significant differences, Log rank<0.05 was used.

TTP/*Zfp36* expression in mammary progenitor cell populations was evaluated in previously reported integrated single-cell RNAseq data sets of post-natal mouse mammary glands (García Solá et al., 2021). This analysis showed that relatively high levels of TTP/*Zfp36* mRNA were detected in basal cell clusters, *Procr*+ mammary stem cells and *Aldh1a3*+ luminal progenitor cells (Figure 2A). A similar distribution in the assembled UMAP was determined for all members of the *Zfp36* gene network that has been previously reported (Canzoneri et al., 2020) (Supplementary Figure S2). Furthermore, focusing specifically on the luminal alveolar populations, we found relevant TTP*/Zfp36* expression levels mostly in progenitor cell clusters (Figure 2B).

In the human breast, most cancer subtypes have shown reduced TTP*/ZFP36* expression compared with normal tissue or “Normal-Like” mammary tumors (Goddio et al., 2012; Canzoneri et al., 2020). However, “Claudin-Low” breast cancers, which present stem-like expression profiles (Fougner et al., 2020), displayed significantly higher *ZFP36* expression levels than Luminal, Her2 and Basal subtypes (Figure 2C). Furthermore, using METABRIC data, we found positive correlations between expression of *ZFP36* and genes associated with the stem-like phenotype, as *TWIST1, TWIST2, SNAI1, ZEB1, ZEB2, ALDH1A and YAP1* (Fougner et al., 2020) in all breast cancer subtypes (Figure 2D). Similar observations were made analyzing TCGA-Pan Cancer data (Supplementary Figures S3A, B) and it was determined that positive correlations of TTP*/ZFP36* expression with those stem-like markers were more significant in primary tumors (all subtypes considered) than in normal tissue (Figure 2E). Besides, analysis of this dataset also revealed that, in spite of clear TTP/*ZFP36* expression downregulation in mammary tumors, high expression of this gene was not associated with better prognosis, considering all breast cancer subtypes, and that overall survival (OS) time was even shorter if only patients with basal-like subtype were considered (Figure 2F). Therefore, we consider that in the human mammary gland, low expression of TTP/ZFP36 may favor tumor development as several oncogenic and inflammatory pathways would be upregulated and therefore may contribute to progression of the disease. On the other hand, breast cancer cells with relatively high levels of this protein would display a stem-like phenotype, which is associated with breast cancer recurrence (Mao et al., 2022).

### 3.3 TTP*/ZFP36* knock-down decreases survival, self-renewal, and repopulation capacity of HC11 mammary stem-like cells

**FIGURE 3:**
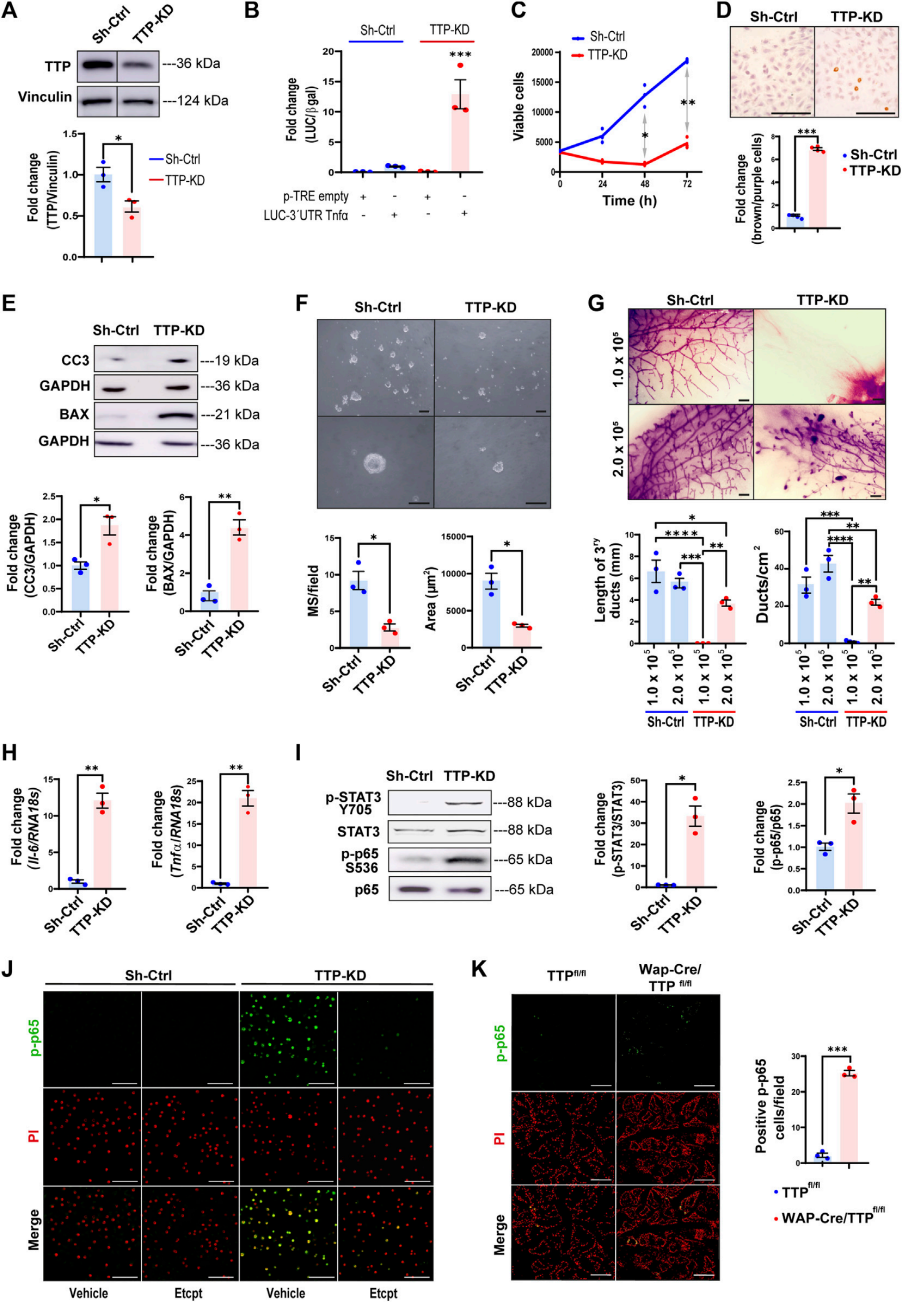
TTP/*Zfp36* knock-down decreases survival and self-renewal, and activation of STAT3 and NFκB pathways in mammary epithelial cells. **(A)** Representative image of TTP/*Zfp36* WB analysis in TTP-KD and Sh-Ctrl cells. Below, quantification plot shows fold change of TTP/Vinculin protein expression ±SEM (T-Student test, *n* = 3, *p < 0.05). **(B)** Luciferase/β-Gal activity ±SEM in Sh-Ctrl (blue) and TTP-KD (red) cells transfected with pTRE-LUC-3′UTR-*Tnfα* or pTRE-control plasmid relatively to Sh-Ctrl cells transfected with pTRE-LUC-3′UTR*-Tnfα* (ANOVA and Tukey test, *n* = 3, ***p < 0.001). **(C)** Metabolic active Sh-Ctrl and TTP-KD cells analyzed by MTS every 24 h for 3 days. Plot shows average number of viable cells ±SEM (two-way ANOVA and Tukey test, *n* = 3, *p < 0.05, **p < 0.01). **(D)** Representative images of Sh-Ctrl and TTP-KD apoptotic cells analyzed by TUNEL assay. On the right, bars show ratio of stained brown (apoptotic)/total nuclei ±SEM fold change of TTP-KD relatively to Sh-Ctrl cells (T-Student test, *n* = 4, ***p < 0.001). **(E)** Representative images of WB analysis of cleaved caspase 3 (CC3) and BAX in Sh-Ctrl and TTP-KD cells. Below, quantification plots showing protein fold changes of CC3/GAPDH ±SEM and BAX/GAPDH ±SEM in TTP-KD relatively to Sh-Ctrl cells (T-Student tests, *n* = 3, *p < 0.05, **p < 0.01). **(F)** Brightfield images of Sh-Ctrl and TTP-KD derived MS 10 days after seeding. OM = 100X (upper images), 400X (lower images). Scale bars = 100µm. Below: plots showing average number per field and diameter ±SEM of MS from Sh-Ctrl and TTP-KD cells (T-Student test, *n* = 3, *p < 0.05). **(G)** Representative images of whole mounted Sh-Ctrl or TTP-KD 1.0 × 10^5^ or 2.0 × 10^5^ cell implants in mammary cleared fat pads (scale bars = 1 mm). On the right, quantification plots of ducts per cm^2^ ± SEM and tertiary ducts length ±SEM (one-way ANOVA and Tukey test, *p* < 0.05, *n* = 3 implanted glands per condition). Significant differences exist between groups with no shared letters. OM: original magnification; MS: mammospheres. **(H)** *Tnfα* and *Il-6* mRNA expression levels showed as fold change ±SEM in TTP-KD relatively to Sh-ctrl cells (T-Student test, *n* = 3, **p < 0.01). **(I)** Representative WB analyses of p-STAT3 (T705) and p-p65 (S536) in Sh-Ctrl and TTP-KD cells (left panel). On the right, bar graphs show fold change of p-STAT3/total STAT3 and p-p65/total p65 ± SEM in TTP-KD relatively to Sh-ctrl cells (T-student test, *n* = 3, *p < 0.05). **(J, K)** Representative images of IF assays for p-p65 (S536) detection in Sh-Ctrl or TTP-KD cells treated with Etanercept or vehicle **(J)** or in Wap-Cre/TTP^fl/fl^ and TTP^fl/fl^ mammary glands on day 15 of their second lactation **(K)**. Nuclei were labeled with Propidium Iodide (PI), after treatment with RNase. Original magnification = ×200. Scale bars = 100 µm. In **(K)**, on the right, quantification of p-p65 positive cells per field ±SEM (T-Student test, *n* = 3, ***p < 0.001). IF: immunofluorescence.

To prove the impact of diminishing TTP levels in mammary progenitor cells, we proceeded to transfect specific *Zfp36* shRNAs complementary to different protein-coding regions in HC11 mammary cells, which display stem-like features (Ferrari et al., 2015; Sornapudi et al., 2018). Although most clones in which TTP/*Zfp36* expression was significantly decreased did not survive more than a few passages, we were able to establish a cell subline, transfected with shRNA 325, named TTP-KD. These cells showed significantly less *Zfp36* mRNA and TTP protein expression than cells transfected with *scrambled* shRNAs (Sh-Ctrl cells) (Figure 3A and Supplementary Figures S4A, B). By immunofluorescence, we have also found weaker nucleic and cytoplasmic TTP staining in TTP-KD compared with ShCtrl cells (Supplementary Figure S4C). Similarly, TTP immunostaining performed in mammary glands of pregnant MG-TTP KO mice displayed less positive cells, and almost complete loss of nuclear staining (Supplementary Figure S4D).

To determine TTP activity in Sh-Ctrl and TTP-KD cells, they were transfected with the pTRE-LUC-3′UTR-*Tnfα* plasmid in which the TTP binding region of *Tnfα* 3′UTR is located downstream Luciferase cDNA (Carballo et al., 1998; Beisang and Bohjanen, 2012). Figure 3B shows that luciferase activity increased about 12-fold, although reduction of TTP*/Zfp36* expression was only around 40%, in TTP-KD compared with Sh-Ctrl cells.

TTP-KD cell line showed reduced viability in different culture conditions (Figure 3C and Supplementary Figure S4E), increased apoptosis (Figure 3D) as well as higher levels of pro-apoptotic BAX and CC3 (Figure 3E). In addition, these cells displayed impaired self-renewal capacity, since they produced smaller and fewer mammospheres than Sh-Ctrl cells in low-attachment plates (Figure 3F). Furthermore, when 1 × 10^5^ TTP-KD cells were implanted into BALB/c inguinal cleared fat pads, no mammary development was observed after 10 weeks, although Sh-Ctrl cells gave rise to fully developed glands. When the number of implanted cells was doubled, both sublines originated mammary ductal networks. However, ducts from 2 × 10^5^ TTP-KD cells did not reach mammary fat pad limits and displayed remaining TEBs, structures that had already disappeared in glands from Sh-Ctrl cells (Figure 3G). Therefore, TTP downregulation resulted in incomplete mammary ductal development *in vivo,* probably due to the impairment of the progenitor cell population. Interestingly, whole mount analysis of 1 × 10^5^ TTP-KD and Sh-Ctrol cell implants performed during lactation revealed that pregnancy allowed development, although incomplete in most cases, and differentiation of the experimental cells (Supplementary Figure S4F). In addition, the ability of TTP-KD cells to differentiate when stimulated with lactogenic hormones in culture has been demonstrated when HC11 TTP-KD cells treated with prolactin and glucocorticoids displayed a relevant increase in STAT5 phosphorylation (Supplementary Figure S4G).

### 3.4 TTP*/ZFP36* downregulation leads to inflammatory cytokine over-expression and activation of stress-associated signaling pathways

TTP-KD cells showed increased *II-6* and *Tnfα* mRNA levels and phosphorylation of transcription factors commonly activated by these cytokines, as STAT3 and NF*k*B p65/RelA, respectively (Berishaj et al., 2007; Kalliolias and Ivashkiv, 2016) (Figures 3H, I). In fact, p65/RelA phosphorylation on S536 was mostly dependent on TNFα overexpression, since this effect was blocked by inhibiting that cytokine with Etanercept (Figure 3J). Importantly, an increase of p-p65 was also observed in the mammary glands of lactating MG TTP-KO compared with control mice (Figure 3K).

**FIGURE 4:**
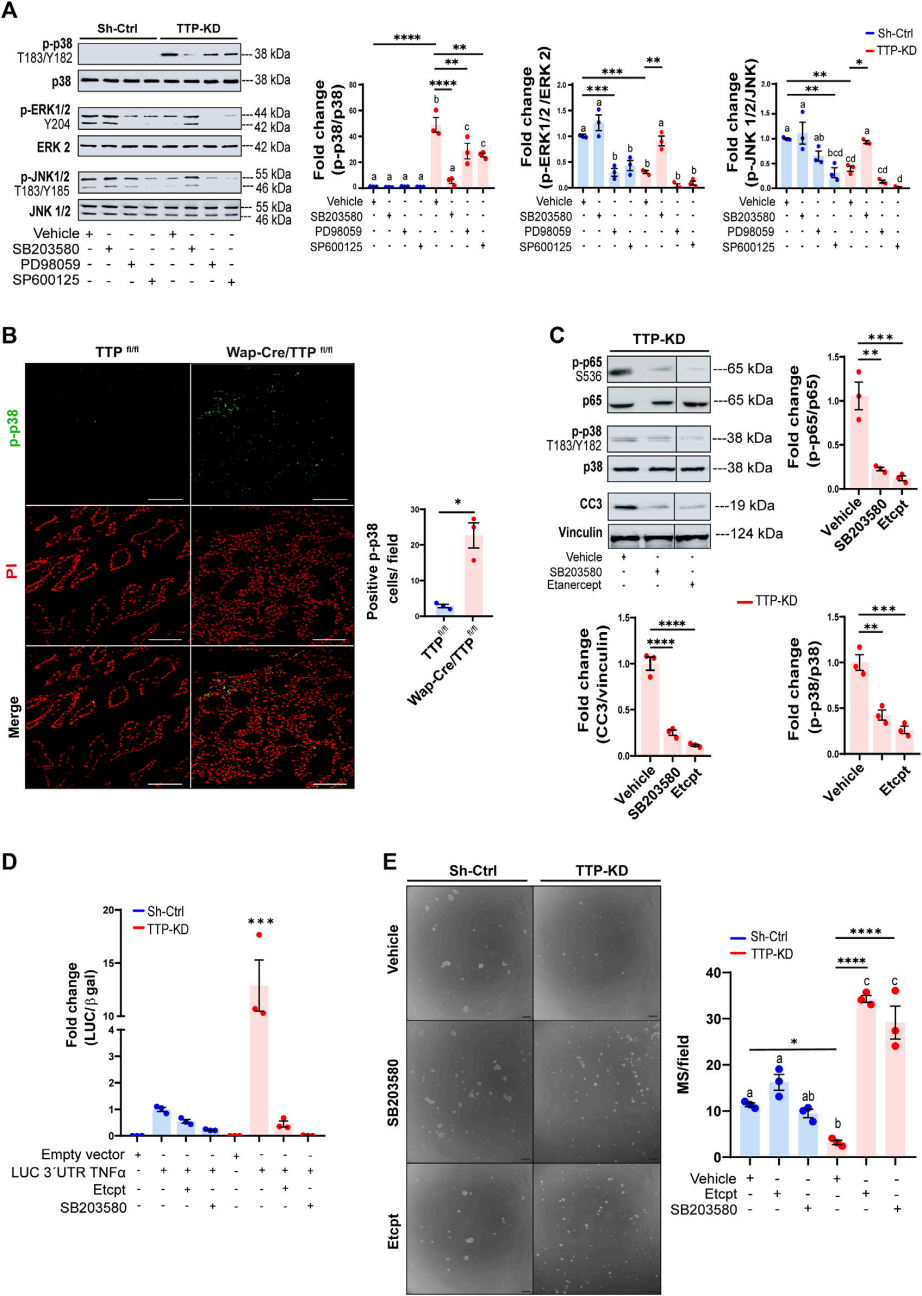
Effects of MAPKp38 activation and TNFα levels on TTP activity and cell renewal on TTP-KD cells. **(A)** Representative WB analyses of p-p38 (T180/Y182), p-ERK1/2 (Y204) and p-JNK1/2 (T183/Y185) in Sh-Ctrl or TTP/KD cells treated with either vehicle, 10μM p38 α and β inhibitor SB203580, 20 μM MEK 1/2 inhibitor PD98059, or 10 μM JNK 1/2 inhibitor SP600125. On the right, quantification plots show fold change of phosphorylated MAPK/total MAPK ±SEM in cells with different treatments relatively to Sh-Ctrl cells treated with vehicle (One way ANOVA and Tukey test, *n* = 3, *p* < 0.05). Significant differences exist between groups with no shared letters. **(B)** Representative images of IF analysis for p-p38 (T180/Y182) detection in Wap-Cre/TTP^fl/fl^ and TTP^fl/fl^ mammary glands at only 15 of their second lactation. Nuclei were labeled with Propidium Iodide (PI). On the right, quantification of p-p38 positive cells per field ±SEM (T-Student test, *n* = 3, *p < 0.05). IF: immunofluorescence. **(C)** Representative WB analyses of p-p65 (S536), p38 (T180/Y182) and CC3, normalized to total p38, total p65 and Vinculin, respectively in TTP-KD cells treated with vehicle, SB203580, or Etanercept. Below, quantification plots show fold change ±SEM relative to TTP-KD cells treated with vehicle. Significant differences exist between groups with no shared letters. **(D)** Bar graph showing fold changes of Luciferase/β-Gal activity ±SEM in Sh-Ctrl and TTP-KD cells transfected with pTRE-LUC-3′UTR-*Tnfα* and treated with 0.03 μg/μL Etanercept, 10 µM SB203580 or vehicle relatively to Sh-Ctrl cells transfected with pTRE-LUC-3′UTR*-Tnfα* vector (ANOVA and Tukey test, *n* = 3, ***p < 0.001). **(E)** Representative images of MS derived from Sh-Ctrl and TTP-KD cells treated with 0.03 μg/μL Etanercept, 10 μM SB203580, or vehicle for 10 days. OM = 100X. Scale bar = 100 µm. On the right, plot shows average number of MS per field ±SEM (One way ANOVA and Tukey contrast, *n* = 3, *p* < 0.05). For **(A, E)**, significant differences exist between groups with no shared letters. For **(A, C, E)** significant differences between specific groups are represented by asterisks (*p ≤ 0.05, **p ≤ 0.01, ***p ≤ 0.001, ****p ≤ 0.0001). MS: mammospheres. OM: original magnification.

Figure 4A shows a drastic increase of constitutive MAPK p38 phosphorylation (p-p38, T183/Y182) while, ERK1/2 and JNK1/2 phosphorylation (Y204 and T183/Y185 respectively) was lower in TTP-KD compared with Sh-Ctrl cells. Interestingly, inhibition of p38, using SB203580, caused a rise of ERK1/2 and JNK1/2 phosphorylation, indicating that p38 overactivation may be responsible for downregulation of ERK1/2 and JNK1/2 activity in TTP-KD cells. On the other hand, ERK and JNK inhibition (by PD98059 and SP600125 treatment, respectively) decreased p-p38 levels in this cell line. Whether that was due to unspecific effects of these pharmacological inhibitors on p38 phosphorylation, or to ERK1/2 and JNK1/2 positive contribution to p38 phosphorylation, remains to be elucidated.

Over-activation of p38 as a consequence of TTP downregulation was also evident in mammary glands of lactating MG-TTP KO females, in which nuclear and cytoplasmic p-p38 labeling was observed. Nuclear localization was prioritized for quantification since it may be related with the pro-apoptotic activity of this enzyme (Wood et al., 2009; Cuadrado and Nebreda, 2010) (Figure 4B).

We determined that secreted TNFα was required for p65/Rel A and p38 phosphorylation as well as caspase 3 activation in TTP-KD cells, as these effects were blocked by Etanercept treatment. Besides, inhibition of p38 phosphorylation reduced p65/RelA phosphorylation and caspase 3 cleavage in these cells (Figure 4C). These results indicated that NFκB activation and apoptosis induction were controlled by p38 activation, which, in turn, depended on TNFα extracellular levels. In addition, Figure 4D shows that Etanercept and SB203580 treatment also prevented the dramatic *Tnfα*-3′UTR stabilization induced by TTP downregulation. Furthermore, inhibiting p38 and TNFα activity not only reversed, but greatly increased mammosphere formation capacity of TTP-KD cells (Figure 4E). Therefore, blocking the TNFα-p38 signaling pathway restores survival of TTP-deficient mammary progenitor cells. However, by analyzing mammosphere sizes, we observed that Etanercept treatment did not reverse proliferative impairment of TTP-KD cells, and that SB203580 inhibited proliferation of Sh-Ctrl cells (Supplementary Figure S4H). These data suggest that mechanisms regulating progenitor cell survival are different from those controlling proliferative capacity of the daughter cells.

## 4 Discussion

Here we show that TTP*/Zfp36* expression is indispensable for controlling TNFα levels in mammary cells, even in the absence of infection, mutations, or autoimmune diseases. This suggests that there is constitutive transcription of the gene encoding that cytokine and its mRNA levels has to be regulated post-transcriptionally to avoid tissue damage. The relevance of post-transcriptional control for TNFα levels in different tissues and embryonic development has been previously pointed out by other authors (Clayer et al., 2020). Our results indicate that in the mammary gland this mechanism is particularly important for stem cells, in which relatively high TTP levels preserve the integrity of the progenitor compartment allowing successive developmental rounds for feeding multiple litters.

Bipotent mammary stem cells (MaSCs) as well as long-lived unipotent stem cells in addition to diverse luminal progenitor subtypes have been identified in mouse and human mammary tissue (Visvader and Stingl, 2014). Particularly, the MG TTP-KO model allowed us to determine the importance of TTP in PI-MECs, which behave as multipotent mammary epithelial progenitors upon transplantation, and as cancer-initiating cells in MMTV-Her2/neu multiparous transgenic mice (reviewed by Wagner and Smith, 2005). These cells are able to form mammospheres in culture and express markers associated with mammary stem cells (Matulka et al., 2007). We have previously shown that TTP/*Zfp36* expression is highly increased during lactation, mainly due to prolactin induction, through Stat5A activation. In addition, we have demonstrated that during lactation, TTP wards off early involution by preventing the untimely increase of local inflammatory factors (Goddio et al., 2018). It has not been determined whether PI-MECs originate from cells expressing WAP that are not terminally differentiated, or whether they arise from differentiated cells that bypass apoptosis during involution or both (Wagner and Smith, 2005). Nevertheless, it can be speculated that TTP/*Zfp36* expression decrease observed in involuting mammary glands (Goddio et al., 2012) would correspond to downregulation of this gene in the differentiated cells, making them susceptible for dying, while PI-MECs would maintain relatively high expression levels, preventing the impairment of this stem-like compartment.

Our analysis of scRNA-seq data sets confirm previously reported observations about conserved co-expression of TTP/*Zfp36* with other early-responsive genes (Canzoneri et al., 2020; Gutierrez et al., 2022). Here we show that coincidence occurs, at least partly, in *Procr+* MaSCs and *Aldh1a3*+ luminal progenitor cells. We have also found expression of this set of genes in some populations of the basal compartment, which might correspond to an “obligatory transitional transcriptional state” described by Gutierrez et al., 2022. We postulate that TTP/*Zfp36* expression may be relevant to maintain these mammary cell populations throughout different periods of the postnatal female mouse life.

Numerous reports have shown that TTP/*ZFP36* downregulation is associated with breast cancer progression and/or treatment resistance (Marderosian et al., 2006; Brennan et al., 2009; Al-Souhibani et al., 2010; Griseri et al., 2011; Goddio et al., 2012; Upadhyay et al., 2013; Canzoneri et al., 2020). However, as pointed out previously (Griseri et al., 2011; Goddio et al., 2012), the relevance of TTP/*ZFP36* mRNA level for breast cancer prognosis is debatable. In fact, using TCGA data, we did not find a negative correlation between high expression of this gene and breast cancer patient survival. Besides, we show that the association between the mammary stem-like phenotype and TTP/*Zfp36* expression can be observed not only in mouse mammary cells, but also in human breast cancer tissue. Interestingly, positive correlation of this gene with different stem-like gene markers was stronger in primary tumors than in breast samples from healthy women, which might be due to a higher proportion of cells that express these markers in neoplastic tissue than in normal mammary epithelium. Therefore, we propose that high TTP levels might be relevant for maintaining the cancer stem cell population in some breast tumors. Furthermore, Wang et al. (2017) have demonstrated that CD8^+^ cytotoxic T lymphocytes (CTLs) are increased in Zfp36^−/−^ mice compared with WT mice. This was due to enhanced production of IL-27 by macrophages in the tumor microenvironment. In agreement with this observation, mammary tumors showed retarded growth in the TTP-KO mice, Therefore, high TTP levels may impair anti-tumor immunity contributing to lower the survival rate of basal breast cancer patients, as displayed in Figure 2F.

The significance of TTP/*Zfp36* expression in the survival of stem-like cells was confirmed by the loss of cleared-fat pad repopulation and mammosphere formation capacity of TTP-KD cell line. These cells, which are still able to express significant amounts of TTP, showed a drastic decrease in their ability to reduce *Tnf*α mRNA probably due to the constitutive activation of p38, which phosphorylates the MAPK-activated protein kinase 2 (MK2) that, in turn, may completely block TTP activity (Tiedje et al., 2016).

Although TTP modulates multiple cytokines, it clearly exerts a dramatic effect on TNFα production by controlling its expression at transcriptional, post-transcriptional and translational level (reviewed by Guo et al., 2017). Furthermore, it has been shown that cachexia, arthritis, and autoimmunity developed by TTP-KO mice were prevented if they were early treated with TNFα antibodies (Taylor et al., 1996). This indicated that the observed phenotype was mostly due to TNFα excessive levels. Here, we show that a pathway triggered by this cytokine would be mostly responsible for apoptosis induction in TTP-KD cells. Similarly, in lactating MG-TTP KO female mice, which also displayed IL-6 over-expression and STAT3 phosphorylation, excessive production, and secretion of TNFα played a key role in cell death induction (Goddio et al., 2018). Nevertheless, high levels of IL-6 and hyper-activation of STAT3 might be also relevant for inducing cell death, as previously reported in involuting mammary glands (Zhao et al., 2002; Hughes and Watson, 2018). TTP activity in modulating STAT3 activation by regulating levels of multiple factors have been reported in different cell types (e.g., Gaba et al., 2012; Jamal Uddin et al., 2013). Interestingly, loss of p63 in the mammary gland, a protein which has also been demonstrated to be essential for the survival and maintenance of PI-MECs, led to epithelial cell death, together with increased activation of the oncostatin M/Stat3 pro-apoptotic pathways (Yallowitz et al., 2014). In addition, TTP may modulate PI-MECs by regulating expression levels of different cytokines, as IL-2 or IL-10 in the mammary gland microenvironment (Ogilvie, R. et al., 2005; Stoecklin, G. et al., 2008).

Our results show that TNFα induces p38 phosphorylation in TTP-KD cells, as reported in other models (Sabio and Davis, 2014; Kalliolias and Ivashkiv, 2016), and that activation of this MAPK leads to mammary cell death, as it has also been previously observed (Wen et al., 2011). Besides, constitutive over activation of p38 may be, at least partially, also responsible for downregulation of ERK1/2 and JNK1/2 phosphorylation. This effect would be similar to what was reported in macrophages by Hall and Davis, 2002.

In summary, we had previously demonstrated that TTP/*Zfp36* expression in the mammary gland is necessary for maintaining lactation and here we show a novel important role for TTP, which is to preserve the progenitor cell compartment. These cells are fundamental for fueling mammary development during puberty as well as throughout each reproductive cycle of female mice. In addition, our results point out the risk involved in long term exposure to high TNFα levels for physiological development, renewal and function of the mammary tissue.

## Data Availability

The original contributions presented in the study are included in the article/Supplementary Material, further inquiries can be directed to the corresponding author.
