# Supplementary material for: Tristetraprolin promotes survival of mammary progenitor cells by restraining TNFα levels
Source: Front Cell Dev Biol. 2024 Jan 11;11:1265475. doi: 10.3389/fcell.2023.1265475 (PMC10808302; doi:10.3389/fcell.2023.1265475)
Supplement: Supplementary file 1 [file DataSheet2.PDF]

Tables

Primer ID	Sequence (5´->3´)
TTP (g)_P1_Fw	GAACCCTCTCTCGATCGGGGATAC
TTP (g/KO) _P2_Rv	GGATGGAGTCCGAGTTTATGTTCCAA
TTP (KO) _P3_Fw	CTGGCTGGAAATGAGAGAGG
TTP (KO) _P4_Rv	CACCCCTTACGCCAGAACTA
WAP-CRE_W003 _Fw	TAGAGCTGTGCCAGCCTCTTC
CRE_C031_ RV	CATCACTCGTTGCATCGACC
MMTV-CRE_Fw	CTGATCTGAGCTCTGAGTG

Table 1: Primer sequences used to genotype transgenic mice strains.

Transgene	Product´s size (pb)	Primers used	Aim
WAP-Cre^{+}	210	W003/C031	Rutinary genotyping
MMTV-Cre^{+}	250	MMTV-CRE/C031	
Zfp36$^{+/+}$	327	P1/P2	
Zfp36$^{fl/+}$	514/327	P1/P2	
Zfp36$^{fl/fl}$	514	P1/P2	
Zfp36$^{+/+}$	683	P3/P2	*Zfp36* KO evaluation
Zfp36$^{fl/+}$	870/683	P3/P2	
Zfp36$^{fl/fl}$	870	P3/P2	
Zfp36Δ	769	P3/P4	

Table 2: Size of genotyping PCR products and combination of primers used.

Primer ID	Sequence (5´-> 3´)	Melting temperature (°C)
Zfp36 (r)_Fw	CCACCTCCTCTCGATACA	60
Zfp36 (r)_Rv	GCTTGGCGAAGTTCACCCA	

Tnfα_Fw	CCACCACGCTCTTCTGTCTACT	60
Tnfα_Rv	GGGTCTGGGCCATAGAACTGAT	
Il-6_Fw	ATCCAGTTGCCTTCTTGGGA	62
Il-6_Rv	CCAGTTTGGTAGCATCCATCA	
Lif_Fw	CAGCGCCAATGCTCTCTTCATT	62
Lif_Rv	ATATTGGTCAGGGAGGCGCT	
Dusp6	CTCGGATCACTGGAGCCAAA	60
Dusp6	GACAGAGCGGCTGATACCTG	60
RNA18s_Fw	GTAACCCGTTGAACCCCACCATT	60
RNA18s_Fw	CATCCAATCGGTAGTAGCG	

Table 3: Primer´s sequences used in RT- qPCR analyses and melting temperatures employed.

Antibody WB	Manufacturer	Catalog number	Concentration	Species
TTP	Lab. Blacksear	------	1:1000	Rabbit
Vinculin	Santa Cruz	Sc-73614	1:5000	Mouse
Cleaved caspase 3	Cell Signaling	9661	1:1000	Rabbit
BAX	Santa Cruz	Sc-493	1:1000	Rabbit
STAT3 total	Cell Signaling	9139	1:1000	Mouse
Phospho-STAT3 (Tyr705)	Cell Signaling	9145s	1:1000	Rabbit
Phospho-STAT5 (Tyr694)	Cell signaling	4322S	1:1000	Rabbit
GAPDH	Santa Cruz	Sc-322233	1:5000	Mouse
p65 total	Cell Signaling	8242	1:1000	Rabbit
phospho-p65 (Ser536)	Cell Signaling	3031	1:1000	Rabbit
ERK2 total	Santa Cruz	Sc-1647	1:1000	Mouse
phospho-ERK ½ (Tyr204)	Cell Signaling	4370	1:1000	Rabbit
JNK ½ total	Cell Signaling	9258	1:1000	Rabbit
phospho-JNK ½ (Thr183/Tyr185)	Cell Signaling	9255	1:1000	Mouse
p38 total	Cell Signaling	92125	1:1000	Rabbit
phospho-p38 (Thr180/Tyr 182)	Cell Signaling	92165	1:1000	Mouse

Table 4: Antibodies used in WB analysis, their manufacturer, catalog number, dilution employed and species in which they were made.

Antibody	Manufacturer	Catalog number	Dilution	Species
Cleaved caspase 3	Cell Signaling	9661	1:200	Rabbit
phospho-p38 (Thr 180/ Tyr 182)	Cell Signaling	4631	1:200	Rabbit

phospho-p65 (Ser 536)	Cell Signaling	3031s	1:200	Rabbit
phospho-STAT 3 (Tyr 705)	Cell Signaling	9145s	1:200	Rabbit
TTP	Lab Balckshear	-------	1:200	Rabbit
TTP	LS Bio	LS-B1572	1:200	Rabbit

Table 5: Antibodies used in IFs and IHCs, their manufacturer, catalog number, dilution employed and species in which they were made.

Antibody	Manufacturer	Catalog number	Dilution	Species
Lineage cocktail	BioLegend	78035	1:50	Mouse
CD24-APC	BioLegend	101813	1:1000	Rat
CD29-FITC	BioLegend	102205	1:100	Mouse

Table 6: Antibodies used in FACS, their manufacturer, catalog number, dilution employed and species in which they were made.
