# Supplementary material for: Tristetraprolin promotes survival of mammary progenitor cells by restraining TNFα levels
Source: Front Cell Dev Biol. 2024 Jan 11;11:1265475. doi: 10.3389/fcell.2023.1265475 (PMC10808302; doi:10.3389/fcell.2023.1265475)
Supplement: Supplementary file 2 [file DataSheet1.PDF]

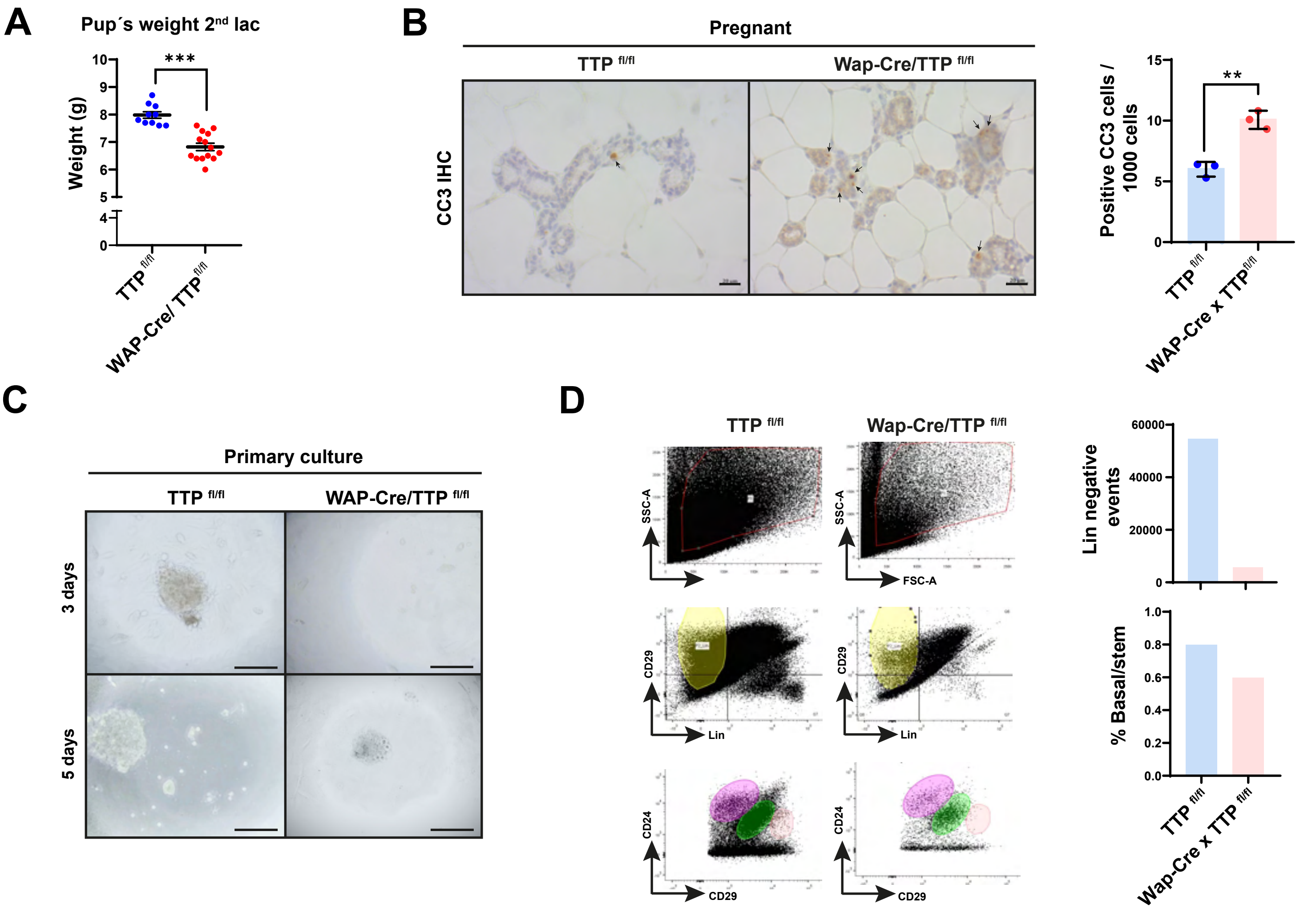

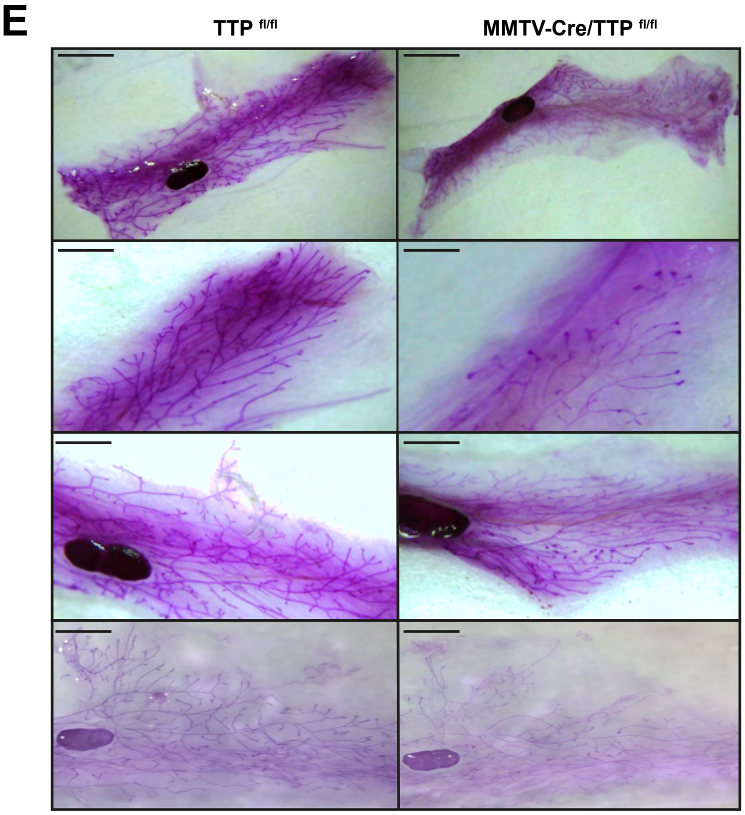

Figure S1. A. Fifteen days-old offspring weight from TTP-fl/fl or Wap-Cre/TTPfl/fl mothers during second lactation (weight ± SEM, n = 10-13, ***p<0.05). **B.** CC3 IHC in mammary glands of pregnant TTPfl/fl or Wap-Cre/TTPfl/fl females. OM 200X, scale bar 20 μM. Arrows indicate positive CC3 nuclei. On the right: positive CC3 every 1000 total cells ± SD. Unpaired t test, ** p ≤ 0.01, n = 3. **C.** Mammary gland primary cultures of post-weaning TTPfl/fl or Wap-Cre/TTPfl/fl female mice. Different times after plating are shown. OM=400X. Scale bars=100 μm. **D.** Flow cytometry analysis of pooled mammary glands from 4 TTPflfl and 4 Wap-CRE/TTPflfl females10 days post weaning with CD24/APC, CD29/FITC and Lineage cocktail/PE conjugated antibodies. On the left: Flow Jo V10 analysis. Cells with normal granularity were gated (P1) in SSC-A vs FSC-A plots. From P1, non-immune cells were selected by gating Lineage (Lin-) cells (P2, yellow gate) by a CD29/FITC vs Lin/PE plots. Lin negative cells (PE negative) quadrants were marked based on a non-stained negative control and PE compensation control. From P2, luminal (CD24high CD29+, pink), basal (CD24+ CD29+, green) and basal enriched in stem cells (CD24+ CD29high, red) populations were gated in CD24 vs CD29 plots. Upper right panel, quantification of P2 events for TTPfl/fl and Wap-CRE x TTPflfl females. Lower right panel, % of basal/stem cells from P2 events for for TTPfl/fl and Wap-CRE x TTPflfl females. **E.** Representative images of whole mounted mammary glands of 4TTP-fl/fl and 4 MMTV-Cre/TTPfl/fl mammary glands nulliparous female mice. Scale bars=1 mm.

*

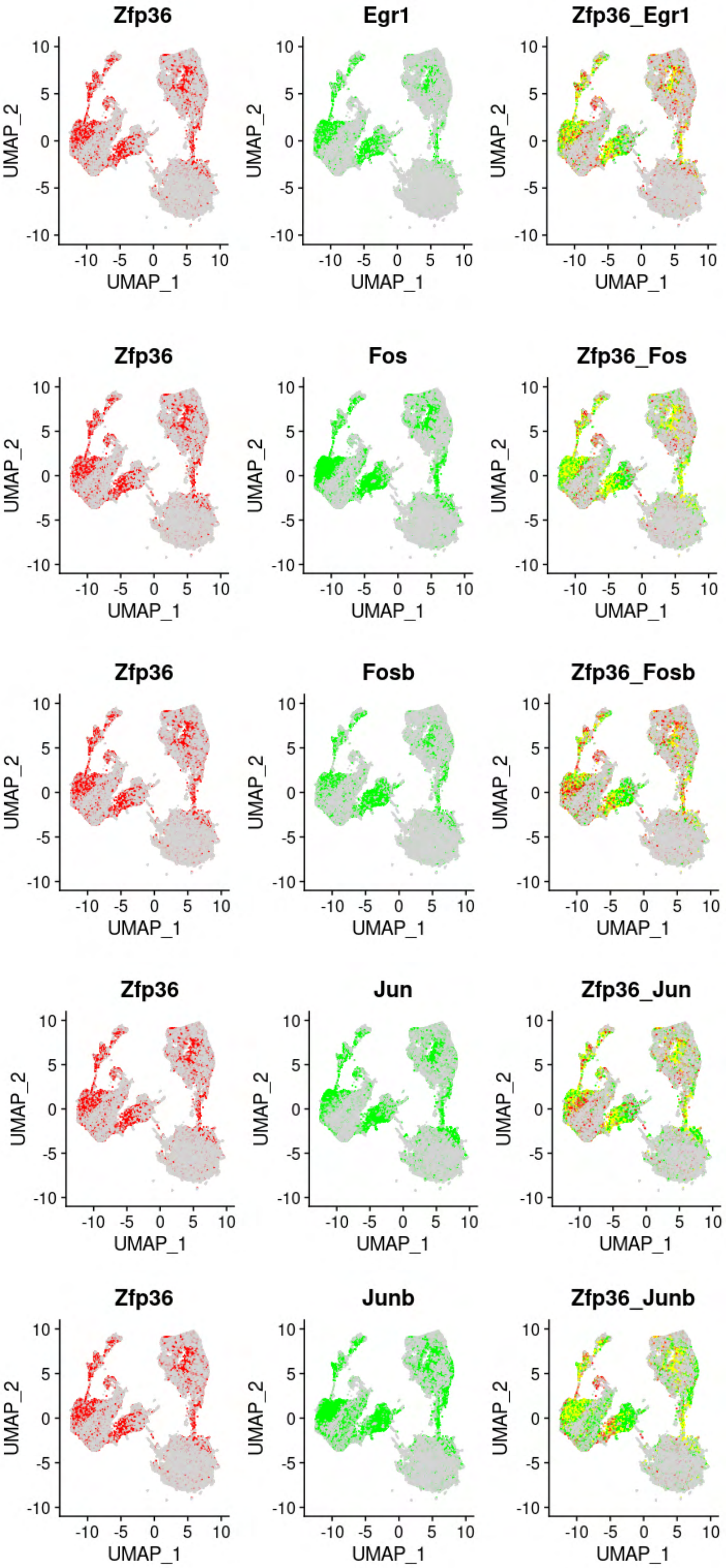

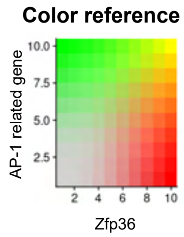

Figure S2. UMAP of post-natal MEC populations displaying scRNAseq relative expression levels of Zfp36 (left panels) in a red scale and Egr1, Fos, Fosb, Jun, Junb AP-1 related genes (central panels) in a green scale. Right panels: Co-expression blend analysis of Zfp36 and each of the AP-1 related genes. Yellow dots depict cells in which both genes are co-expressed, as shown in the color reference.

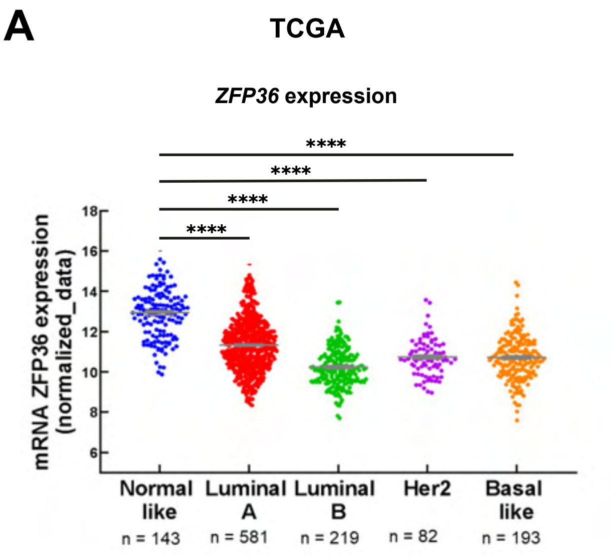

B TCGA

	ZFP36 All subtypes	Normal like	Luminal A	Luminal B	HER2	Basal like
TWIST1	0,43 ****	0,43 ****	0,43 ****	0,09 ns	0,36 ***	0,23 **
TWIST2	0,61 ****	0,40 ****	0,53 ****	0,39 ****	0,43 ****	0,44 ****
SNAI1	0,38 ****	0,36 ****	0,39 ****	0,33 ****	0,23 *	0,31 ****
ZEB1	0,41 ****	0,30 ***	0,33 ****	0,20 **	0,08 ns	0,24 ***
ZEB2	0,44 ****	0,30 ***	0,38 ****	0,23 ***	0,18 ns	0,28 ****
ALDH1A1	0,53 ****	0,40 ****	0,51 ****	0,19 **	0,27 *	0,23 **
YAP1	0,30 ****	0,12 ns	0,18 ****	0,14 *	0,00 ns	-0,16 *

Color scale: 1, 0, -1

Figure S3. A. Expression of ZFP36 mRNA (log2 values) in breast cancer molecular subtypes according to TCGA database. Kruskal Wallis and Dunn's tests were performed. Significant differences exist between groups with no shared letters ($p<0.05$). **B.** Expression correlation between ZFP36 and genes related to stem/progenitor behavior comparing different breast cancer subtypes according to TCGA database. On the right of each graph, color scale (red: positive, blue: negative) indicates correlation level. Spearman's ranked correlation test was applied (*p<0.05, **p<0.01, ***p<0.001, ****p<0.0001).

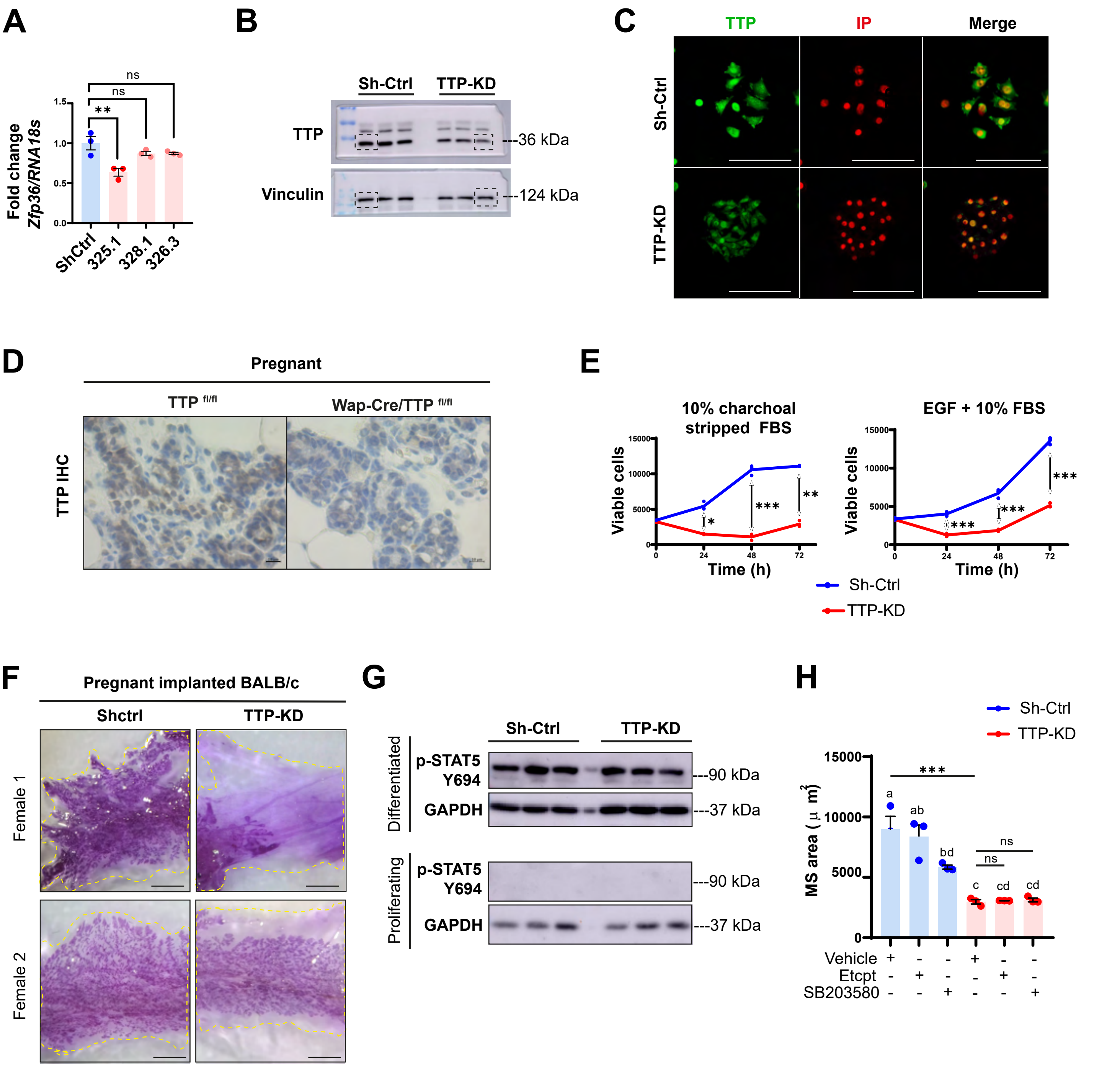

Figure S4. A. Zfp36 mRNA expression in HC11 clones stably transfected with shRNAs complementary to different Zfp36 coding regions relatively to HC11 cells transfected with scrambled ShRNA (Sh-Ctrl cells). Values are shown as fold change of Zfp36/RNA18s . **B.** Western Blot analysis of TTP and Vinculin expression in ShCtrl and TTP-KD cells were. Bands shown in Fig. 3A are into dashed boxes. **C.** Representative images of immunofluorescence (IF) showing TTP expression in Sh-Ctrl and TTP-KD cells. Nuclei were stained with propidium iodide (PI). OM=200X, scale bar=100 µm. **D.** TTP immunohistochemical (IHC) staining in mammary glands of pregnant TTP-fl/fl or Wap-Cre/TTPfl/fl females. OM 400X, scale bar = 10 mM. **E.** MTS analysis of Sh-Ctrl and TTP-KD cells growing in 10% charcoal stripped FBS media or 10% FBS media supplemented with 2.5 ng/mL Epidermal Growth Factor (EGF). Plot shows average number of viable cells ± SEM (two-way ANOVA and Tukey test, n=3, *p<0.05, **p<0.01) sampled every 24 h for 3 days. Significant differences exist between groups with no shared letters. **F.** Examples of whole mounted cleared fat-pads implanted with either ShCtrl or TTP-KD cells from lactating BALB/c female mice. Scale bar = 0.5 cm. Dashed yellow lines show fat pad limits. **G.** Western blot analysis of pSTAT5 Y694 and GAPDH from proliferating and differentiated Sh-Ctrl and TTP-KD cells. H. Area covered by mammospheres of Sh-Ctrl and TTP-KD cells treated with either Etanercept or SB203580. Bars display Area ± SEM (n=3). Significant differences exist between groups with no shared letters. For specific groups of interest, the p-value is represented with asterisks (ns = p > 0.05, *p ≤ 0.05, **p ≤ 0.01, ***p ≤ 0.001, ****p ≤ 0.0001). One way ANOVA and Tukey contrast.

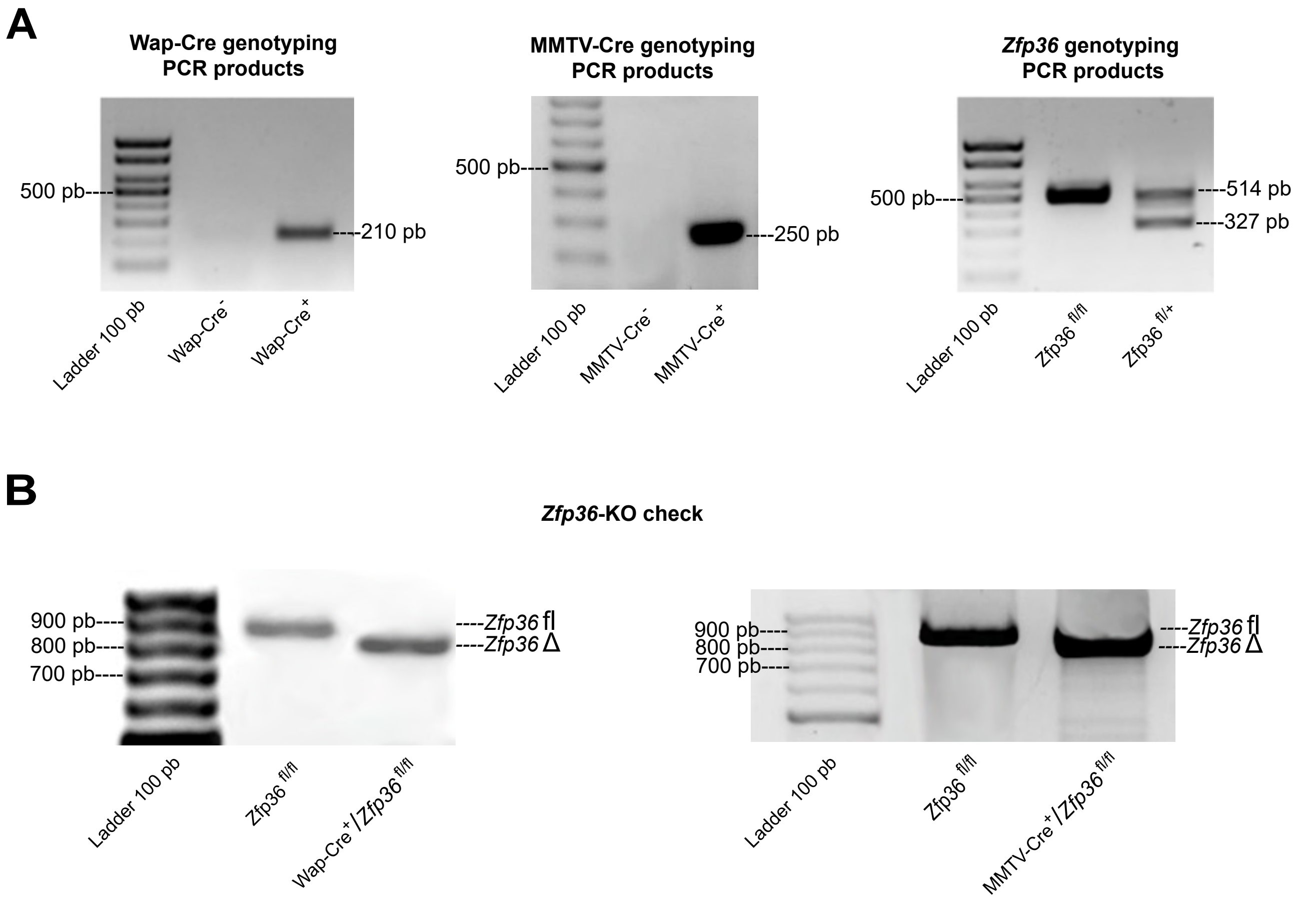

Figure S5. A. Representative images of agarose gels showing Wap-Cre (left), MMTV-Cre (center) and Zfp36 (right) genotyping DNA-PCR products. Product size is shown on the right of each photograph. Mouse genotypes are indicated below each lane. In all cases mouse tail DNA was used. **B.** Representative images of agarose gels showing Zfp36-KO DNA-PCR products from MECs of control (Zfp36fl/fl mice) or TTP-KO mice: Wap-Cre/Zfp36fl/fl mice on the right, and MMTV-Cre/Zfp36fl/fl mice on the left. PCR from control mouse DNA produces a 870 bp band (from floxed Zfp36 alleles) while mammary DNA from TTP-KO mice produces a 769 bp band (recombined Zfp36 or Δ alleles). The primers used for all these PCR analyses are specified in Suppl. Table 1 and product sizes in Suppl. Table 2.
